# Genomic analysis of breed composition and population structure in Montana composite cattle

**DOI:** 10.1007/s00335-026-10275-8

**Published:** 2026-09-07

**Authors:** Caroline Assis Almeida, Rafael Espigolan, Rachel Santos Bueno Carvalho, Elisângela Chicaroni de Mattos, Victor Breno Pedrosa, Gabriela Giacomini, Fernando Baldi, José Bento Sterman Ferraz

**Affiliations:** 1 Faculty of Animal Science and Food Engineering, University of São Paulo, Pirassununga, SP Brazil; 2 Federal University of Santa Maria, Palmeira das Missões, Santa Maria, RS Brazil; 3 Neogen Corporation, Lincoln, NE 68504 USA; 4 International Association of Montana Breeders, Mogi Mirim, SP Brazil

## Abstract

The Montana composite was developed in Brazil from crosses between *Bos indicus* and *Bos taurus* and structured into four biological types: Zebu (N), adapted taurine (A), British taurine (B), and continental taurine (C). This study aimed to characterize the genetic diversity and population structure of the Montana composite using genomic data through principal component analysis (PCA), admixture analysis, and Wright’s F_ST_ statistic. The PCA revealed a clear separation between *Bos indicus* and *Bos taurus* groups, with Montana animals distributed in an intermediate position. The first two principal components explained 69.48% and 3.45% of the total variation, respectively. Supervised admixture estimates indicated a predominance of taurine contribution, with type A accounting for 34.47%, 52.64%, and 51.71% at K = 4, 9, and 11, respectively. Increasing the ancestry resolution refined the contribution of individual founder breeds without changing the overall predominance of taurine ancestry. Comparisons between breed proportions obtained from pedigree and genomic data revealed significant differences, for most biological types and ancestry models (*P* < 0.001), indicating that realized breed composition deviates from theoretical expectations. Estimates of genetic differentiation confirmed greater divergence between Zebu and taurine groups, as well as reduced distances among populations sharing common ancestry. Specific relationships were identified between the composite and some of its founder breeds, particularly Belmont Red, Senepol, and Tuli. Overall, the results demonstrate that the Montana composite has a complex genomic structure, with genomic ancestry varying according to the resolution adopted and differing from pedigree-based expectations.

**Supplementary Information:**

The online version contains supplementary material available at https://doi.org/10.1007/s00335-026-10275-8.

## Introduction

Production animals have been subjected to intense natural and artificial selection processes, targeting specific traits. In addition to selection, crossbreeding has combined complementary traits from different breeds by mating genetically distinct populations (Paim et al. 2022a). In this context, the Montana composite was developed in Brazil, in 1994, from the crossing of different taurine and zebu breeds, to bring together, within a single genetic group, desirable traits from each of them, such as hardiness, sexual precocity, reproductive efficiency, carcass quality, and adaptability to tropical and subtropical conditions (Ferraz et al. 1999a; Oliveira et al. 2024).

This composite is based on the crossing of four biological types: Zebu (N), adapted taurine (A), British taurine (B), and continental European taurine (C) (Ferraz et al. 1999a). Although the Montana program has defined requirements for the proportions of these four biological types, the specific breeds within each type are not fixed. Therefore, breed composition may vary according to producers’ decisions and farm conditions, since the program uses an open system that allows the continuous inclusion of animals recognized as genetically superior (Ferraz et al. 1999b).

However, for an animal to be considered a Montana composite, it must meet some requirements related to its breed composition. Among these criteria, the animal must have at least three distinct breeds, with minimum proportions of 12.5% for type A and 25% for the sum of types N and A. The maximum allowed limits are 37.5% for type N, 87.5% for type A, and 75% for types B and C (Santana et al. 2012). Understanding population structure and genetic diversity is fundamental for animal breeding programs, as it guides effective strategies for selection and reproductive management, as well as for the conservation of genetic variability in herds. Describing these aspects allows the understanding of genetic mechanisms related to environmental adaptation and the preservation of genetic resources (Xia et al. 2021).

Traditional pedigree based methods do not account for variability arising from Mendelian segregation, whereas incorporating genomic data enables more accurate estimates of genetic parameters, minimizes possible pedigree errors, and better represents Mendelian sampling (VanRaden 2008; Legarra et al. 2009). Thus, population structure analysis should be an integral part of planning and revisioning breeding programs and conservation strategies for both purebred and crossbred populations (Mulim et al. 2022). This study aimed to analyze population structure, breed ancestry, and genetic differentiation in Montana composite cattle at the breed level using genomic data. Principal component analysis (PCA), admixture analysis, and Wright’s F_ST_ were performed to obtain a clear view of the patterns of admixture and genetic differentiation in the composite population.

## Materials and Methods

### Animals and their sources

**Fig. 1:**
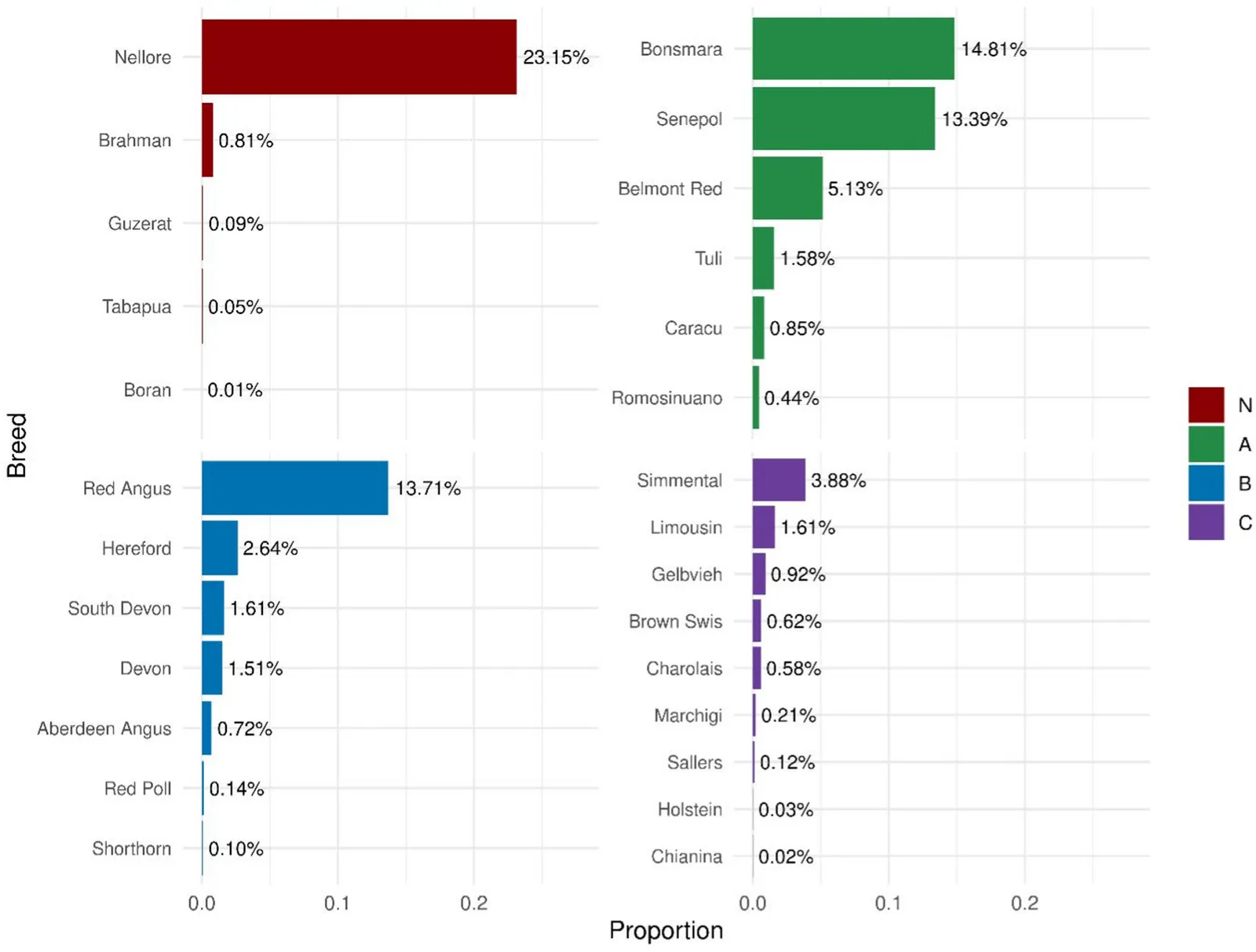
Contribution of the main breeds within each biological type based on the pedigree of the Montana composite. N: Zebu; A: adapted taurine; B: British taurine; C: continental European taurine

To understand the population structure of the Montana composite, a pedigree-based analysis (Fig. 1) was performed to identify the main breeds contributing to its formation. The pedigree database comprised 371,596 animals, including 7,561 sires and 121,817 dams, and covered up to 11 generations. A total of 6,669 animals were inbred, with an average inbreeding coefficient of 0.08%. The pedigree information was used to determine the theoretical breed composition of the composite population and to identify the breeds with the greatest contributions within each biological type. The breeds with the greatest contribution within each biological type were Nellore (type N), Bonsmara and Senepol (type A), Red Angus (type B), and Simmental (type C).

The dataset included 3,892 Montana composite animals and 15 breeds involved in its formation: Aberdeen Angus, Belmont Red, Bonsmara, Boran, Brahman, Guzerat, Hereford, Limousin, Nellore, Red Angus, Senepol, Simmental, Sindi, Tabapua, and Tuli (Table 1). In addition to the data already available at the Animal Breeding and Biotechnology Group (GMAB) of the Faculty of Animal Science and Food Engineering (FZEA) at the University of São Paulo (USP), additional information was obtained from the International Montana Breeders Association, the Animal Genetics and Breeding Program, and the Neogen corporation. In total, 19,947 animals were considered, including 15,623 *Bos indicus* animals and 432 *Bos taurus* animals.

**Table 1:** Summary of cattle samples by population, respective breeds, biological types, number of animals and number of SNPs

Breed	Biological type	Number of animals	Number of SNPs	Number of autosomal SNPs
Aberdeen angus	B	50	95,256^1^	89,795
Belmont red	A	50	95,256^1^	89,795
Boran	N	50	54,791^2^	51,975
Bonsmara	A	20	95,256^1^	89,795
Brahman	N	105	54,791^2^	51,975
Guzerat	N	60	54,791^2^	51,975
Hereford	B	49	95,256^1^	89,795
Limousin	C	49	95,256^1^	89,795
Montana	Composite	3,892	54,791^2^	51,975
Nellore	N	14,998	54,791^2^	51,975
Red angus	B	48	95,256^1^	89,795
Senepol	A	72	95,256^1^	89,795
Simmental	C	50	95,256^1^	89,795
Sindi	N	341	54,791^2^	51,975
Tabapua	N	69	54,791^2^	51,975
Tuli	A	44	54,791^2^	51,975

^1^ GeneSeek^®^ Genomic Profiler Bovine 100 K (Neogen); ^2^GeneSeek^®^ Genomic Profiler Bovine 50 K GGP 50 K (Neogen);* N* Zebu,* A* adapted taurine,* B* British taurine,* C* continental European taurine.

**Fig. 2:**
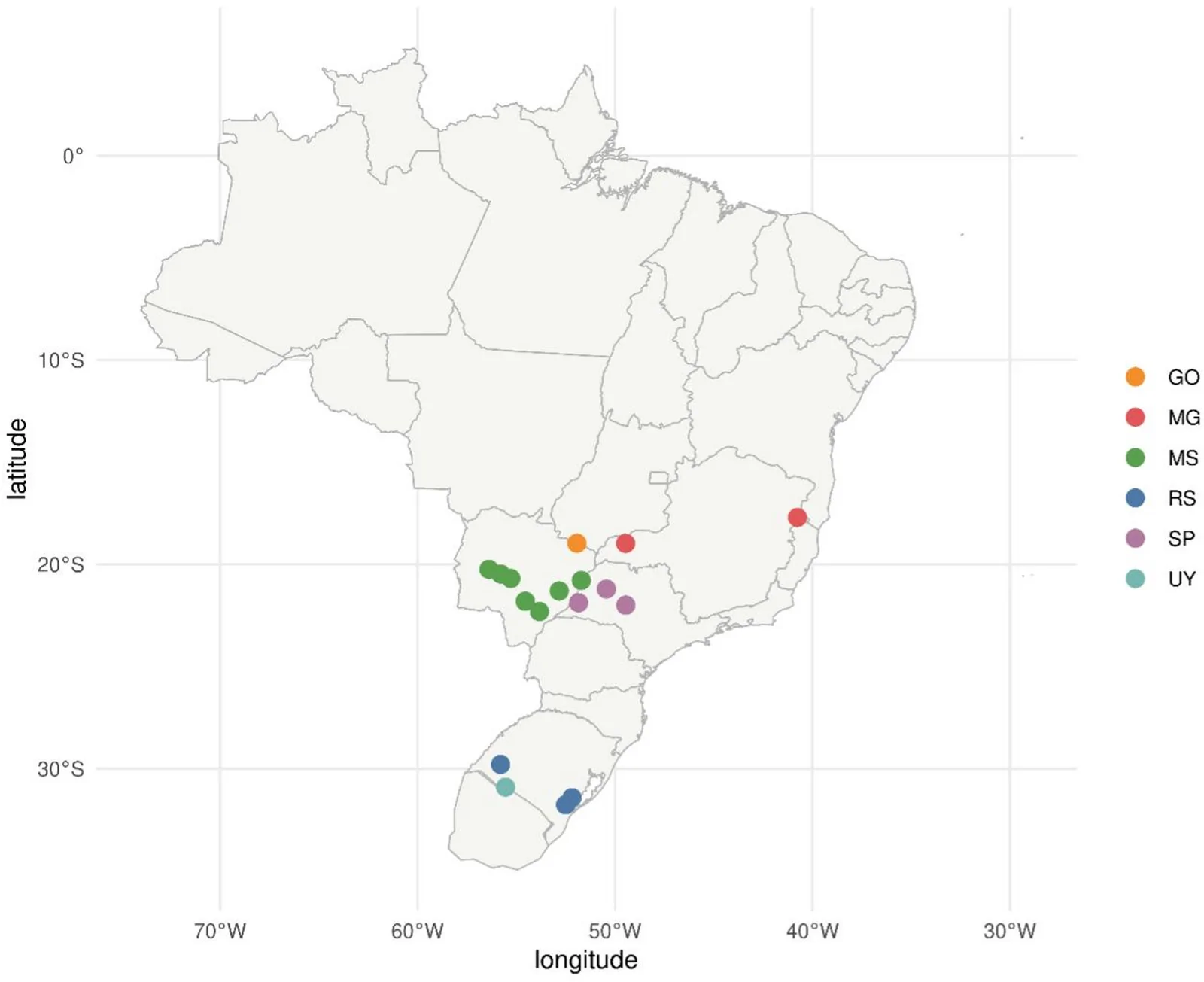
Geographic distribution of the sampled Montana composite herds used for genomic analyses. GO: Goiás; MG: Minas Gerais; MS: Mato Grosso do Sul; RS: Rio Grande do Sul; SP: São Paulo; UY: Uruguay

The Montana composite animals in this study originated from 18 commercial herds across Brazil’s main beef-producing regions, including the states of Goiás, Mato Grosso do Sul, Minas Gerais, Rio Grande do Sul, and São Paulo, as well as one herd from Uruguay (Fig. 2).

### Genotypes and quality control

The number of SNPs per breed varied due to the different densities of the genotyping platforms used. Initially, a consistency check was performed within each breed, removing animals with SNP call rate < 90%, as well as clones, sex chromosomes, and mitochondrial markers. After this procedure, two panels containing 95,256 and 54,791 SNPs remained, as shown in Table 1. To harmonize the genotype datasets obtained from different platforms (GeneSeek^®^ Genomic Profiler Bovine 100 K and GGP Bovine 50 K), all SNP genomic coordinates were updated to the ARS-UCD1.2 bovine reference genome assembly. Marker orientation and allele coding were standardized to the Forward strand according to the array manifest files provided by the manufacturer (Neogen Corp., Lincoln, NE, USA). SNPs were matched based on marker IDs and chromosome and physical positions. Ambiguous A/T and C/G single nucleotide polymorphisms, markers with mismatched chromosomal positions between manifests, and non-overlapping SNPs were excluded. Following this harmonization step, a unified subset of 17,619 autosomal SNPs shared between the two platforms was retained.

This approach was necessary to combine datasets across different genotyping arrays without imputing missing markers, thus avoiding potential imputation bias in crossbred populations. Although this intersection reduced the overall marker density, the retained panel showed mean minor allele frequencies (MAF) above 0.20 and high expected heterozygosity across both taurine and indicine reference breeds (Table S1), indicating that the shared subset retained sufficient genetic variation for population structure, PCA, and ancestry analyses.

Quality control (QC) analyses for the autosomal SNP set were performed using the PREGSF90 software (AGUILAR et al., 2014). The quality control procedure aimed to ensure high genotyping accuracy and retain high-quality biallelic SNPs across populations by removing rare and poorly call-rated variants. The following QC criteria were applied: removal of SNPs with minor allele frequency (MAF) < 0.05 (1,333), exclusion of SNPs with call rate < 0.90 (120), exclusion of SNPs showing extreme departure from Hardy–Weinberg equilibrium (*P* < 1 × 10⁻⁴) (85), and removal of monomorphic SNPs (0). After QC, 16,081 SNPs were retained for subsequent analyses.

### Genetic structure analysis

#### Principal component analysis

PCA based on genotype data was also used to evaluate the population structure of the Montana composite population and to compare it with the breeds that compose it. The analyses were conducted to confirm the relationships among populations, as well as to identify possible substructures within populations. To define covariances among animals, a genomic relationship matrix (**G**) was constructed using only SNPs common to all breeds, according to Method I proposed by VanRaden (2008):

$$\:\mathbf{G}=\frac{\mathbf{Z}{\mathbf{Z}}^{\mathbf{{\prime\:}}}}{2\sum\:{\mathrm{p}}_{\mathrm{i}}(1-\:{\mathrm{p}}_{\mathrm{i}})}$$

where the centered genotype matrix ($\:\mathbf{Z}$) was obtained by subtracting matrix **P** from the genotype matrix **M**, with  **P** = 2($\:{\mathrm{p}}_{\mathrm{i}}$ – 0.5), in which p_i_ is the allele frequency at locus i. Three independent PCAs were performed: the first including all animals; the second excluding the Nellore population; and the third excluding both the Nellore and Montana composite populations. These scenarios were adopted because PCA projections can be strongly influenced by imbalances in sample size among populations, which may introduce bias in the distribution of samples and distort the principal component space (McVean 2009). The exclusion of populations with larger sample sizes changed the variance explained by the principal components, therefore, the three analyses were used to better represent the population structure.

#### Admixture analysis

A maximum likelihood model implemented in ADMIXTURE 1.3 software (Alexander et al. 2009) was used to estimate breed proportions of Montana composite animals. Considering the large imbalance in the number of Nellore animals relative to the other breeds composing the composite, the number of Nellore individuals was reduced to 985 based on criteria of genetic contribution to the herd (number of progeny and use in mating), to minimize bias in admixture estimates. After this reduction, the genotype dataset comprised a total of 5,936 animals (N = 1,612; A = 186; B = 147; and C = 99).

Additionally, a new genotype quality control was performed using PREGSF90 (Aguilar et al. 2014). SNPs were excluded based on the following criteria: call rate < 0.90 (244), MAF < 0.05 (219), monomorphic SNPs (0), Hardy–Weinberg equilibrium deviation (104), and linkage disequilibrium pruning with a threshold of r² > 0.5 (1,389). After these steps, 15,663 SNPs remained for admixture analyses.

Unsupervised admixture analyses with K values (number of inferred ancestral populations) ranging from 1 to 25 were evaluated using cross-validation. Analyses were conducted with parallel execution (4 processor threads), using the block relaxation algorithm as the point estimation method and the quasi-Newton algorithm to accelerate convergence. The accuracy of estimates was assessed using 200 bootstrap resampling and 25 cross-validation (CV) partitions. Although no single K value corresponding to a clear minimum cross-validation error was identified (Fig. S1), the CV profile showed a gradual decrease followed by a plateau at higher K values, indicating increasing model resolution without a single dominant partition. This pattern may reflect the complex genetic structure of the Montana composite, resulting from contributions of multiple founder breeds. Therefore, K = 4, 9, and 11 were considered as biologically defined scenarios based on the biological types and founder breed history.

Thus, ADMIXTURE software (Alexander et al. 2009) was used in supervised analyses to estimate ancestry proportions based on predefined reference populations. Reference animals corresponded to genotyped individuals from the respective founder breeds, based on their known breed classification, with the number of animals for each breed presented in Table 1, except for Nellore, for which the number of individuals was reduced as described previously. This approach improves the interpretability of ancestry estimates in admixed populations by anchoring allele frequency estimation to biologically defined groups, reducing uncertainty associated with closely related or weakly differentiated founder breeds (Alexander and Lange 2011).

The number of reference populations analyzed was K = 4, 9, and 11, representing different hierarchical levels of genetic resolution within the Montana composite. For K = 4, breeds were grouped into four predefined biological categories representing major functional breed groups contributing to the composite population: N = Boran, Brahman, Guzerat, Nellore, Sindi, and Tabapua; A = Belmont Red, Bonsmara, Senepol, and Tuli; B = Aberdeen Angus, Hereford, and Red Angus; and C = Limousin and Simmental.

For K = 9, indicine breeds (Nellore, Brahman, Guzerat, Tabapua, and Sindi) were grouped into a single reference population, reflecting their shared ancestry and relatively limited genetic differentiation, while the Boran breed was treated as an independent indicine lineage due to its distinct African origin. British taurine breeds remained grouped at this level due to their close genetic relationship and shared breeding history. The remaining founder breeds were treated as separate reference populations to preserve breed-specific resolution.

For K = 11, a finer subdivision of taurine breeds was introduced, with British breeds separated into individual reference populations. This refinement was motivated by the historical formation of the Montana composite, in which Red Angus has played a more prominent role compared to the other British breeds, allowing the assessment of differential contributions within this group. This analysis allowed the characterization of the genomic breed composition of the composite population and its comparison with estimates obtained from pedigree data. To investigate whether genomic ancestry varied among production regions, admixture results were also visualized according to the state of origin of each herd.

For comparisons between pedigree and genomic estimates at K = 9 and K = 11, genomic components were aggregated according to their corresponding biological types before comparison with pedigree-based proportions. At K = 9, the N category comprised the grouped indicine component and Boran, whereas A comprised Tuli, Belmont Red, Bonsmara, and Senepol, and C comprised Limousin and Simmental. At K = 11, the individual British components (Aberdeen Angus, Red Angus, and Hereford) were combined to represent biological type B, while N, A, and C were obtained by aggregating their respective genomic components according to the biological type classification.

Pedigree-based and genomic ancestry proportions were compared using paired Wilcoxon signed-rank tests, with the same 3,892 animals used as paired units. Twelve comparisons were performed for the four biological types at K = 4, 9, and 11. P-values were adjusted for multiple comparisons using the Benjamini–Hochberg procedure to control the false discovery rate. Rank-biserial correlation was calculated as a measure of effect size, with 95% confidence intervals, to quantify the magnitude of differences between pedigree-based and genomic estimates. A significance level of 5% was adopted.

#### Genetic relationship and differentiation

The level of genetic divergence among populations was evaluated using Wright’s fixation index (F_ST_) estimated by the method of Weir and Cockerham (1984) using PLINK 1.9 software (Chang et al. 2015). F_ST_ is commonly used to identify genetic differentiation among populations, as it provides an estimate of how genetic variability is partitioned among populations (Wright 1951; Weir and Cockerham 1984). Estimates were obtained from pairwise comparisons between populations using the weighted multilocus estimator (weighted F_ST_), which combines variance components across loci and corrects for differences in sample size among groups (Weir and Cockerham 1984).

According to Wright (1951), F_ST_ values between 0 and 0.05 indicate little or no genetic differentiation, values between 0.05 and 0.15 indicate moderate differentiation, values between 0.15 and 0.25 are considered high, and values above 0.25 indicate very high genetic differentiation. However, these values should be interpreted with caution, as the magnitude of F_ST_ may vary depending on the SNP set used and the number of informative markers available in each analyzed population (Willing et al. 2012). In the context of admixture studies, F_ST_ allows the measurement of genetic distance between ancestral populations, supporting the interpretation of population structure and genetic relationships among the evaluated breeds (Lawson et al. 2018).

## Results

### Principal component analysis

**Fig. 3:**
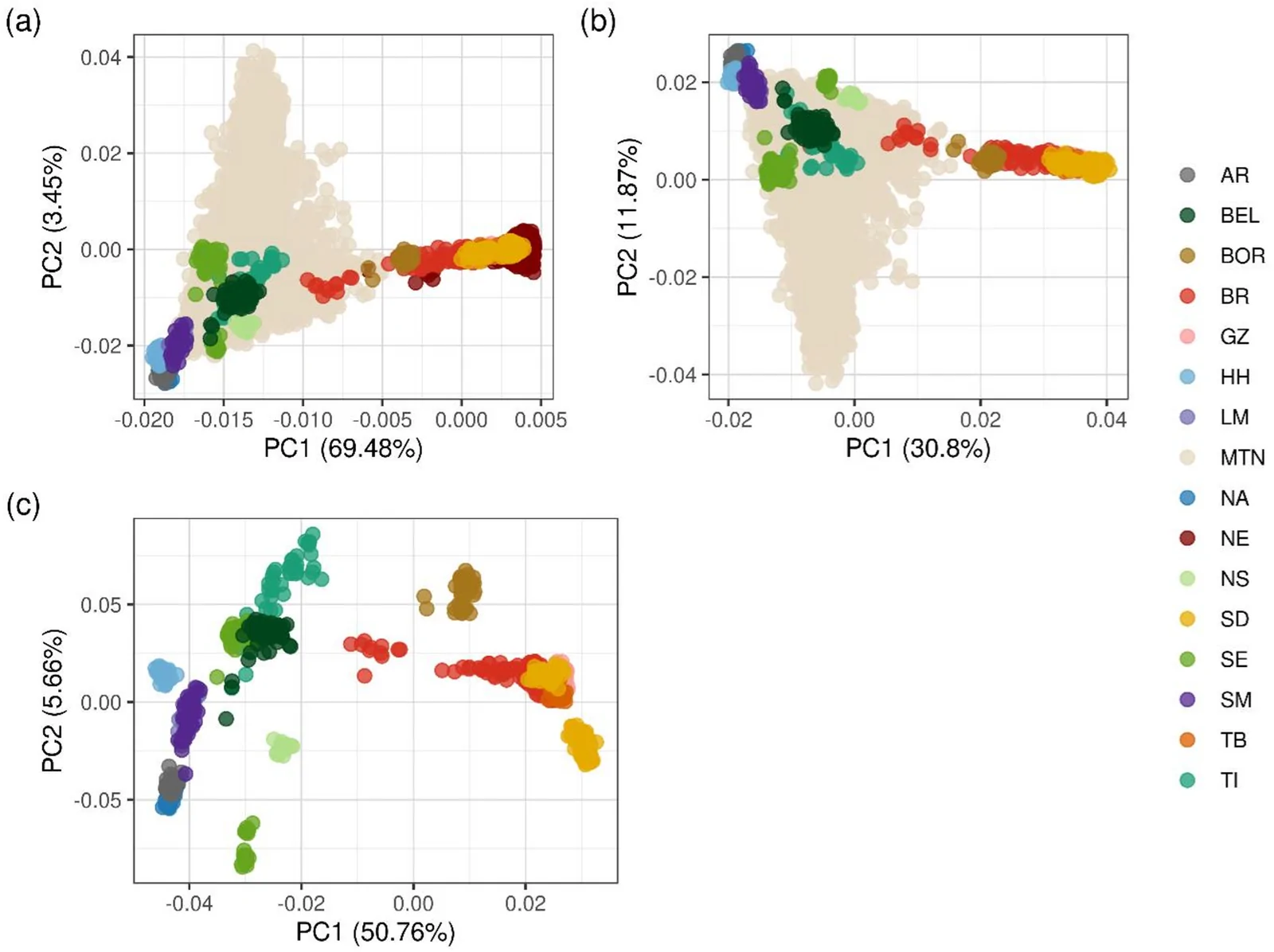
Principal component analysis (PCA) based on SNPs for evaluating the population structure of the Montana composite.** a**: PCA considering all evaluated populations;** b**: PCA after removing the Nellore population;** c**: PCA after removing the Nellore and Montana composite populations. AR: Red Angus; BEL: Belmont Red; BOR: Boran; BR: Brahman; GZ: Guzerat; HH: Hereford; LM: Limousin; MTN: Montana composite; NA: Aberdeen Angus; NE: Nellore; NS: Bonsmara; SD: Sindi; SE: Senepol; SM: Simmental; TB: Tabapua; TI: Tuli

The three PCAs indicated that the first two components visually captured the population structure of the Montana composite and the breeds that compose it, allowing the identification of clustering among the different biological types (Fig. 3). In Fig. 3a, considering all populations, the distance between clusters of points in the multivariate space defined by the principal components (PC) reflected the average genetic distance among the evaluated populations. As expected, PC1, which explained 69.48% of the total variance, accounted for the main genetic separation, highlighting the high divergence between *Bos indicus* and *Bos taurus* groups.

Zebu breeds (Nellore, Guzerat, Sindi, and Brahman) formed a compact and well-defined cluster, while European taurine breeds (Aberdeen Angus, Red Angus, Hereford, Limousin, and Simmental) clustered consistently at the opposite extreme of this axis. Montana composite animals were distributed in an intermediate position between these groups, reflecting their multibreed genetic composition. PC2, although explaining a smaller proportion of the total variance (3.45%), allowed the identification of additional genetic variation within populations.

In Fig. 3b, after removing the Nellore population, PC1 and PC2 explained 30.80% and 11.87% of the total variance, respectively. The overall clustering pattern was similar to that observed in the analysis including all populations, with the main genetic relationships among the biological types maintained. In Fig. 3c, after removing the Nellore and Montana populations, a greater level of detail in the genetic structure among the founder breeds was observed. In this analysis, PC1 explained 50.76% of the total genetic variance, while PC2 explained 5.66%, allowing the identification of distinct clusters among European taurine, tropical-adapted taurine, and Zebu breeds. Tropical-adapted breeds, such as Tuli, Belmont Red, Bonsmara, and Senepol, were positioned intermediately between European taurine and Zebu breeds.

### Admixture analysis

**Fig. 4:**
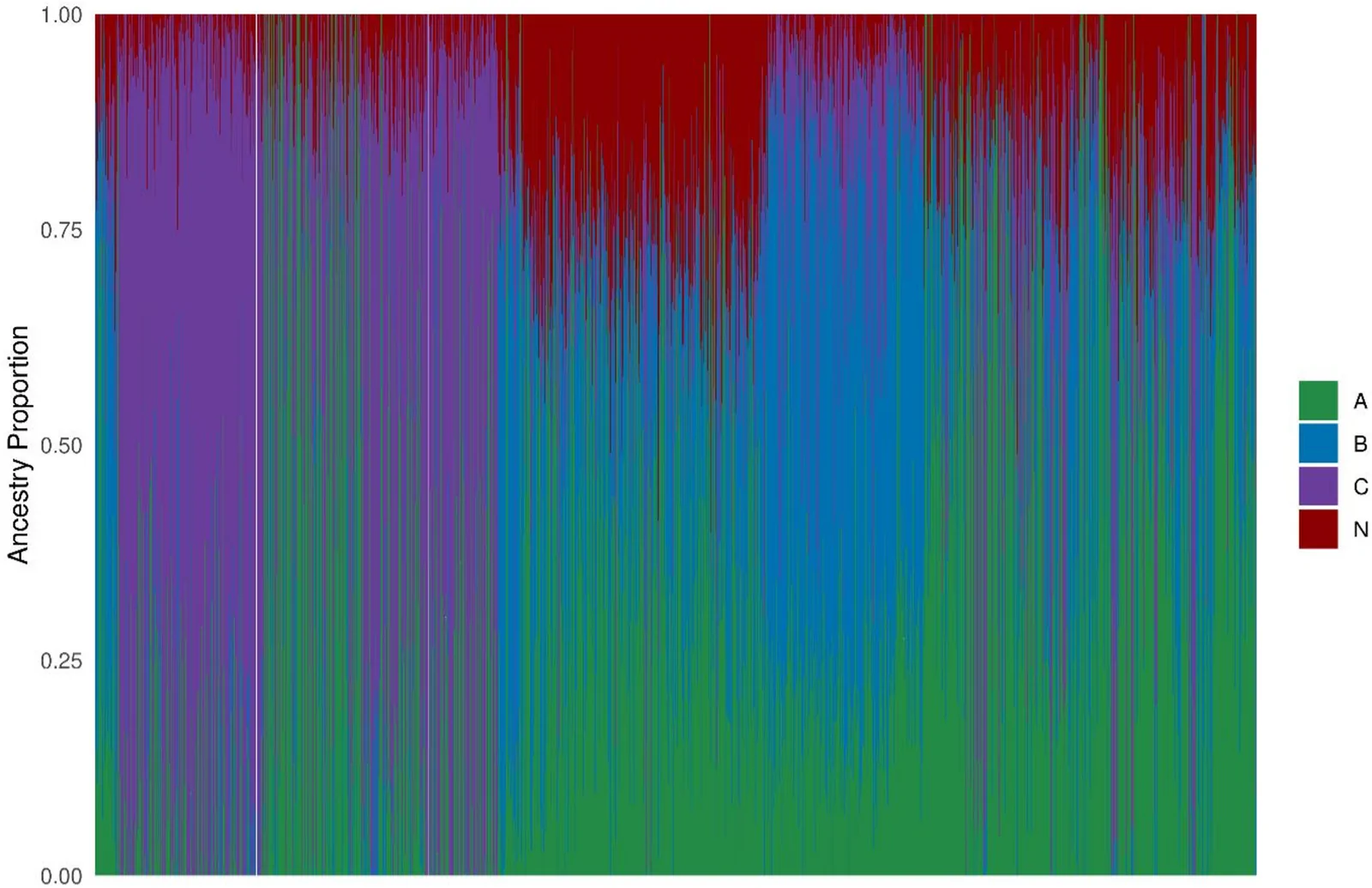
Ancestry proportions of the biological types of the Montana composite estimated by supervised admixture analysis (K = 4). A: adapted taurine; B: British taurine; C: continental European taurine; N: Zebu

**Fig. 5:**
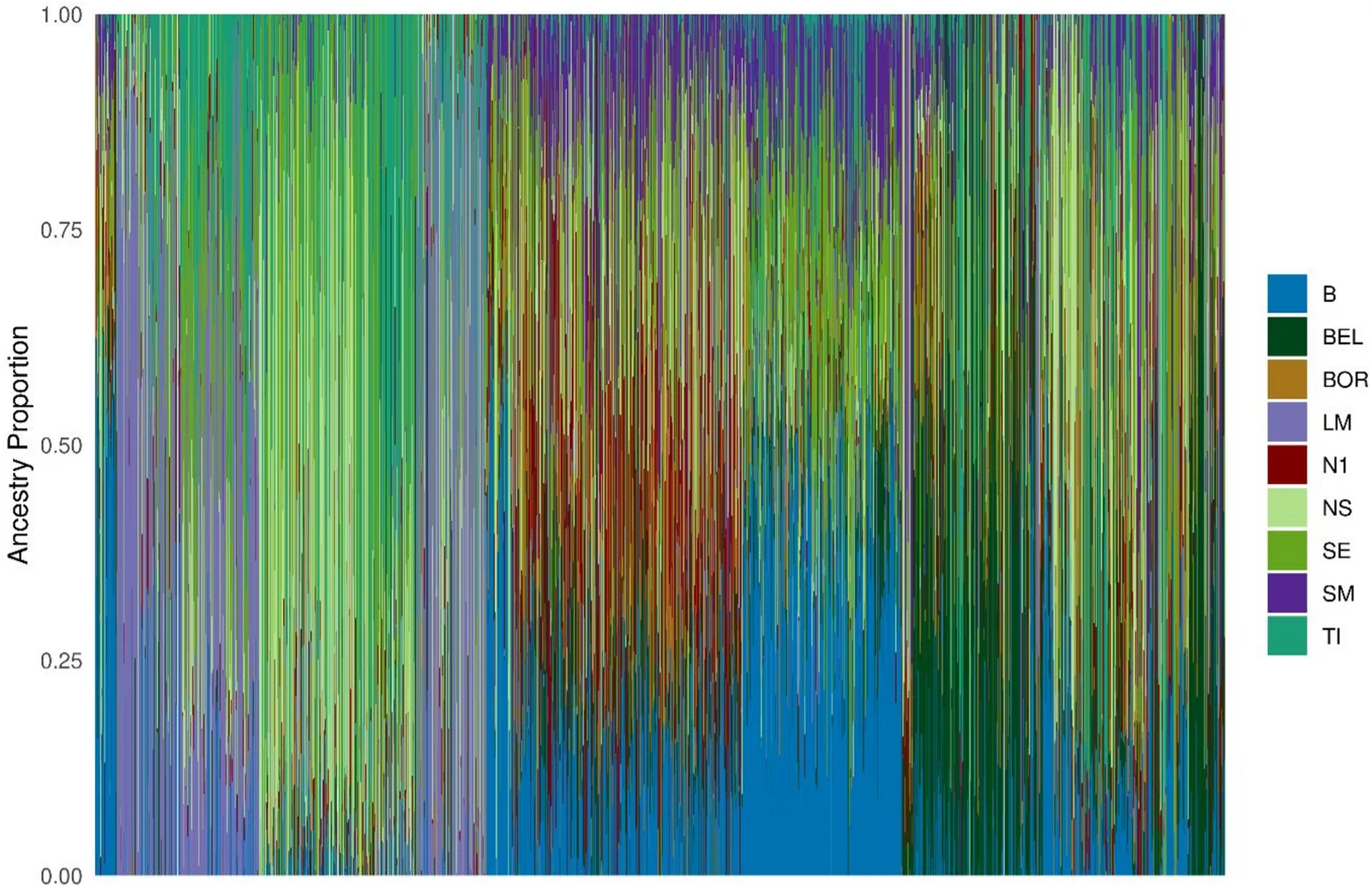
Ancestry proportions of the breeds involved in the formation of the Montana composite estimated by supervised admixture analysis (K = 9). B: British taurine; BEL: Belmont Red; BOR: Boran; LM: Limousin; N1: grouped indicine breeds (Nellore, Brahman, Guzerat, Tabapua; and Sindi); NS: Bonsmara; SE: Senepol; SM: Simmental; TI: Tuli

**Fig. 6:**
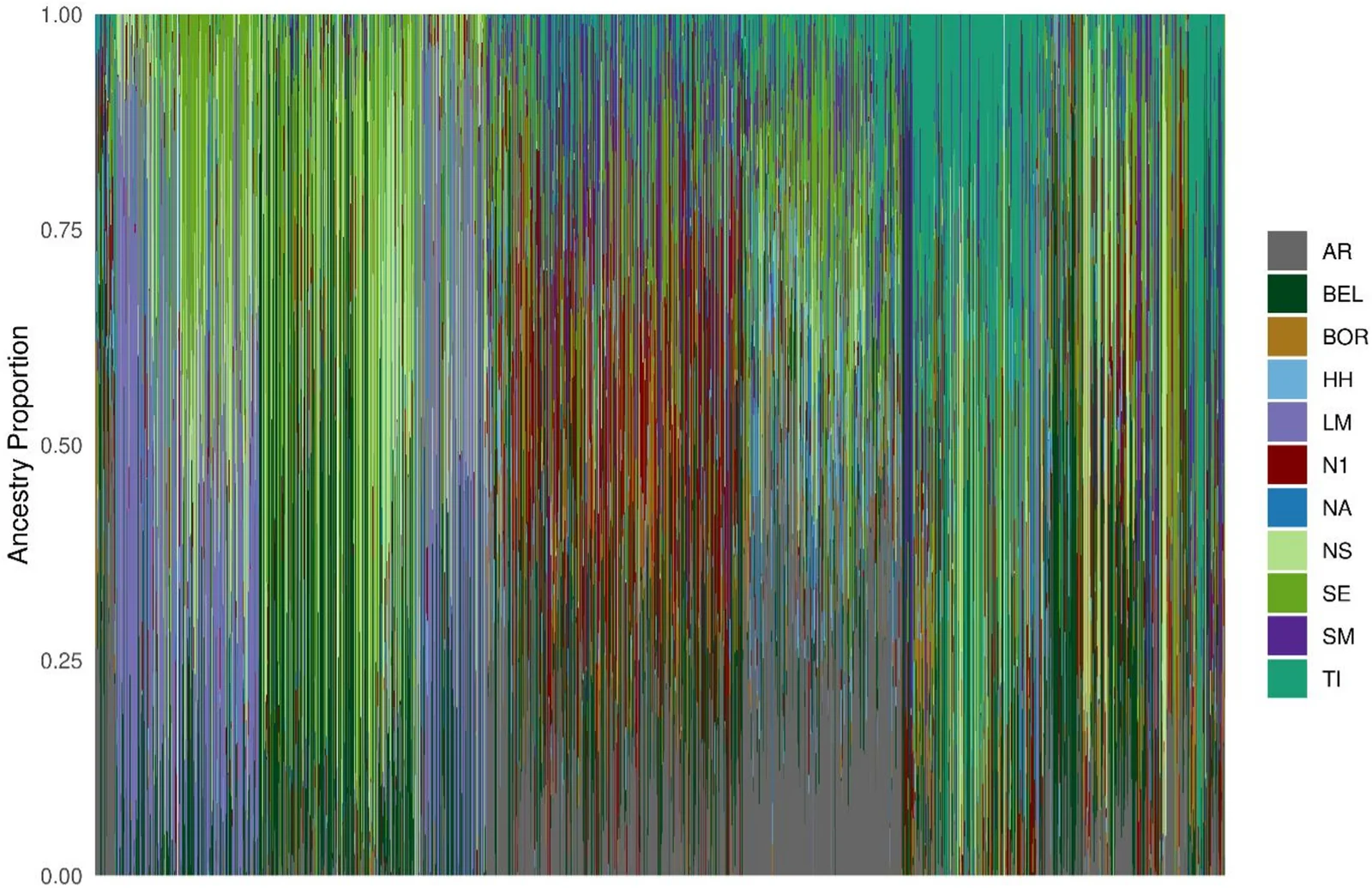
Ancestry proportions of the breeds involved in the formation of the Montana composite estimated by supervised admixture analysis (K = 11). AR: Red Angus; BEL: Belmont Red; BOR: Boran; HH: Hereford; LM: Limousin; N1: grouped indicine breeds (Nellore, Brahman, Guzerat, Tabapua; and Sindi); NA: Aberdeen Angus; NS: Bonsmara; SE: Senepol; SM: Simmental; TI: Tuli

Figures 4, 5 and 6 present the ancestry estimates obtained through supervised admixture analyses, considering K = 4, 9 and 11. In Fig. 4, it is observed that composite animals show varying contributions from the four biological types. In general, there is a predominance of biological types A (34.47%) and C (31.19%) in the genomic composition of Montana animals, followed by type B (21.93%), while type N showed the lowest average contribution (12.40%). These estimates suggest a greater relative contribution of types A and C under the considered model.

At Fig. 5 (K = 9), a finer characterization of the founder populations was observed. The highest average ancestry proportions were associated with Bonsmara (15.21%), Senepol (14.44%), the grouped British taurine (13.23%), Tuli (12.05%), Limousin (11.13%) and Belmont Red (10.94%). The grouped indicine component (N1) accounted for 7.61% of the genomic composition, while Boran contributed 6.54%. Simmental showed the lowest average contribution among the taurine founder populations (8.87%). These estimates indicate a higher representation of taurine components in the Montana composite under this model, while retaining contributions from the different founder groups.

Figure 6 (K = 11) presents the ancestry estimates with the biological type B represented by its individual founder breeds. The highest average breed contributions were observed for Belmont Red (17.22%), Senepol (13.55%), Red Angus (11.72%), Bonsmara (11.01%), and Limousin (10.78%). The grouped indicine component (N1) and Boran contributed 7.81% and 6.00% of the genomic composition, respectively. Among the British breeds, Aberdeen Angus and Hereford exhibited lower average contributions, representing 3.00% and 2.00% of the genomic composition, respectively. At K = 11, the British taurine component was represented by its constituent breeds, with Red Angus showing the highest average contribution among them.

**Fig. 7:**
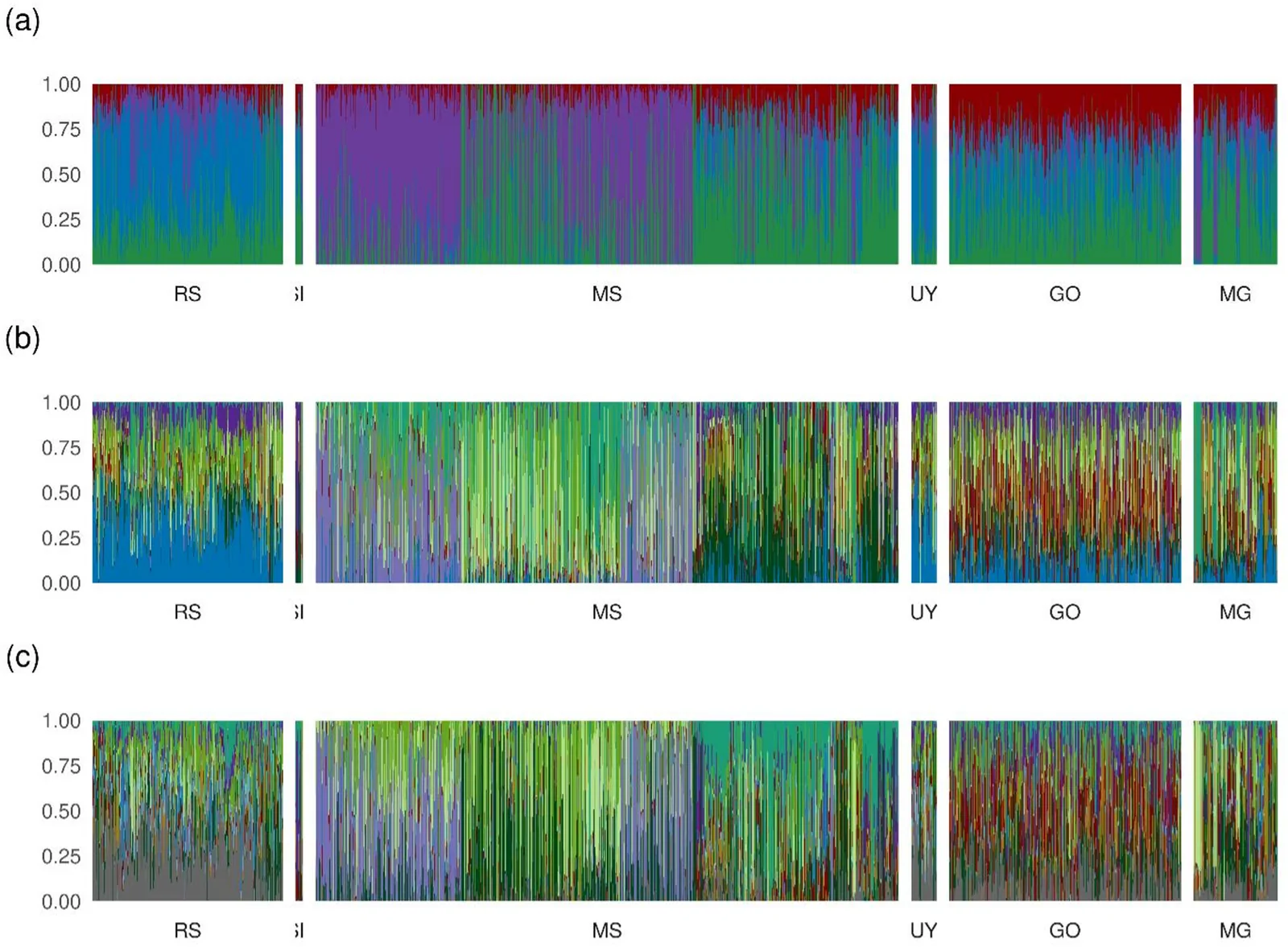
Supervised admixture analysis of Montana composite animals grouped by herd geographic location. (a) K = 4; (b) K = 9; (c) K = 11. RS: Rio Grande do Sul; SP: São Paulo; MS: Mato Grosso do Sul; UY: Uruguay; GO: Goiás; MG: Minas Gerais

The admixture plots sorted according to the predominant biological type or breed are presented in Fig. S2 - S4. When individuals were grouped according to the geographic origin of the herds (state or country), variation in ancestry proportions was observed among regions and within some regions (Fig. 7). Although all regions comprised animals with admixed genomic backgrounds, the relative contributions of the ancestral components varied among herds.

At the biological type level (K = 4), variation in ancestry composition was observed among regions. Animals from RS and UY showed the highest average proportions of type B (51.40% and 53.08%, respectively), whereas MS presented the greatest contribution of type C (48.23%). SP and MG showed the highest average proportions of type A (42.58% and 42.04%, respectively). GO exhibited the highest indicine contribution (25.84%) together with a more balanced distribution among the four biological types.

At K = 9 and K = 11, the ancestry estimates showed variation among regions in the contributions of the individual founder breeds represented in each model. Within the British group, Red Angus represented the largest proportion of British ancestry in all evaluated regions. Its contribution was particularly pronounced in RS and UY, where it accounted for approximately 71.6% and 62.0% of the total British ancestry, respectively. Continental taurine ancestry was predominantly associated with Simmental in most regions, whereas Limousin was the main continental contributor in MS. GO also presented the highest contribution of indicine breeds among the evaluated regions.

#### Breed composition pedigree versus genomic

**Fig. 8:**
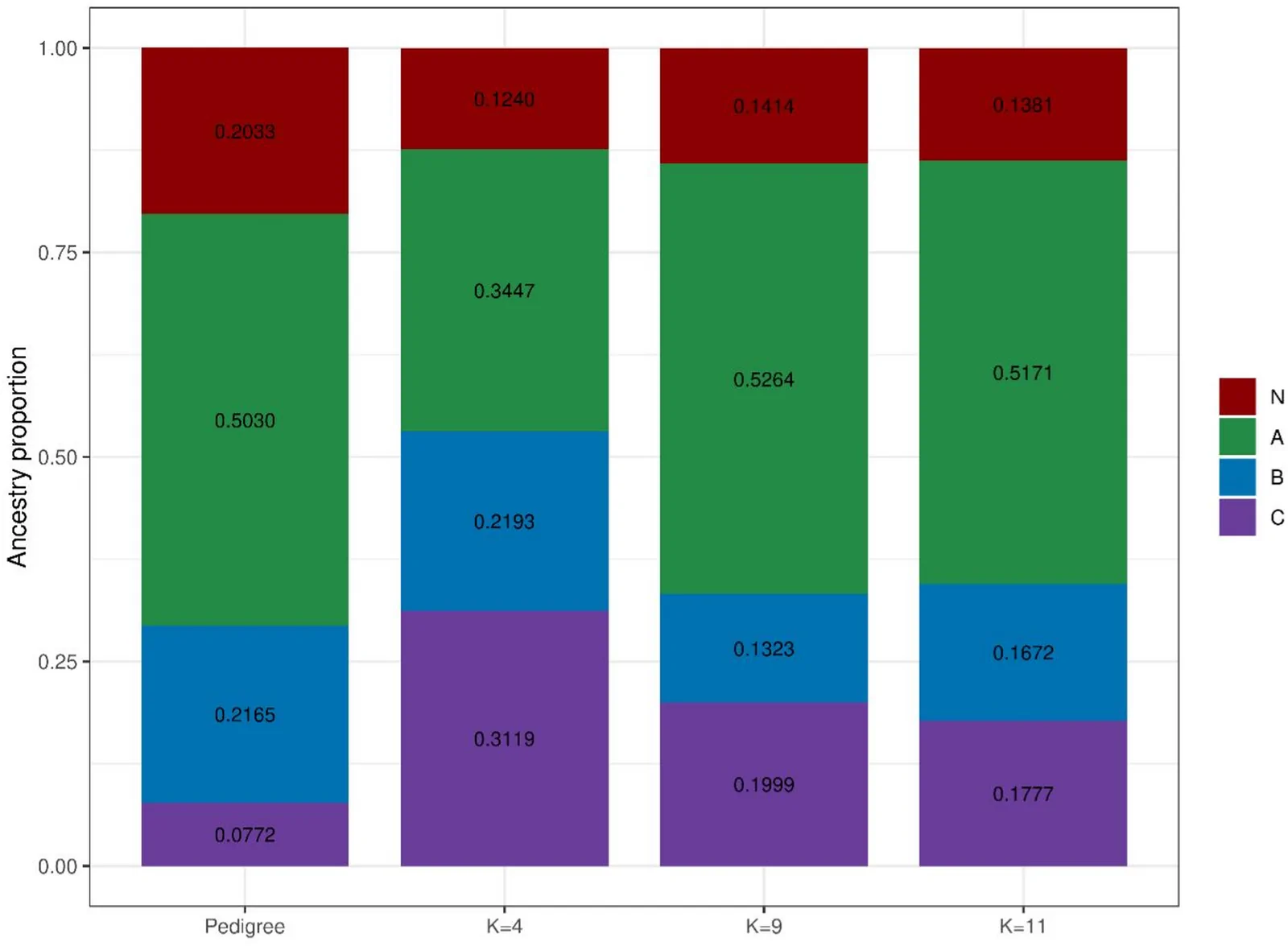
Average ancestry proportions of the biological types of the Montana composite estimated from pedigree information and genomic data (K = 4, 9 and 11).N: Zebu; A: adapted taurine; B: British taurine; C: continental European taurine

Figure 8 presents the comparison between the average proportions of biological types in the Montana composite estimated from pedigree information and genomic data, considering K = 4, 9 and 11. Overall, both approaches indicated a predominance of taurine ancestry; however, differences in the magnitude of breed contributions were observed depending on the method and level of genomic resolution. At K = 4, statistically significant differences were observed for types N, A, and C (FDR-adjusted *P* < 0.001), with rank-biserial correlations of 0.8364, 0.5129, and − 0.5455, respectively, whereas no difference was observed for type B (*P* = 0.389; *r* = 0.016). At K = 9 and K = 11, differences between pedigree and genomic estimates were observed for all four biological types (FDR-adjusted *P* < 0.001), although the magnitude of the effects varied among components (Table S2). When comparing average ancestry proportions at the breed or grouped-breed level (Table S3), pedigree-based estimates showed higher Zebu contributions relative to genomic estimates, whereas adapted taurine breeds, particularly Tuli and Belmont Red, showed lower pedigree-based estimates.

### Genetic relationship and differentiation

**Fig. 9:**
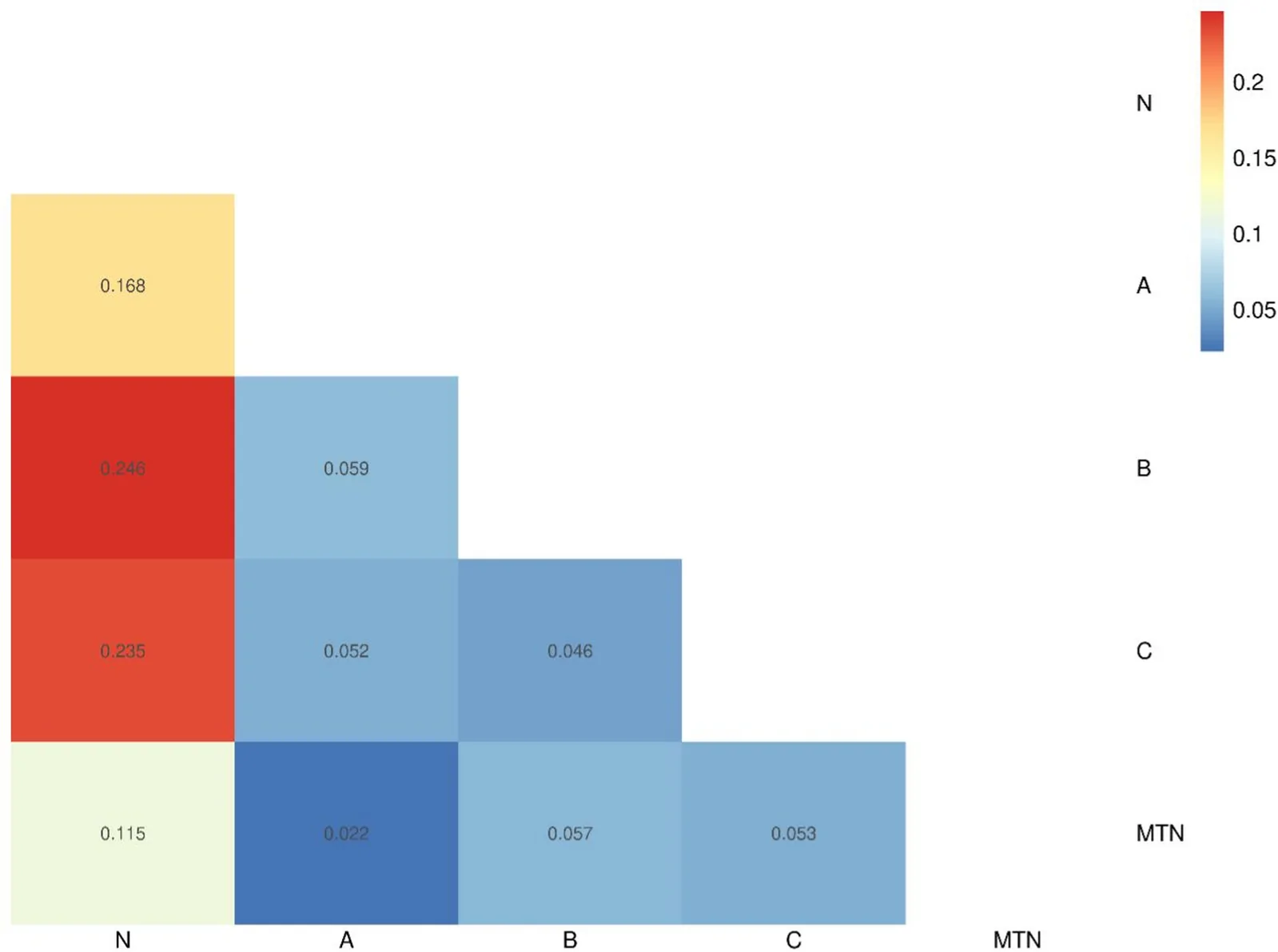
Heatmap of genetic relationships among different biological types and the Montana composite, estimated from pairwise F_ST_ values. N: Zebu; A: adapted taurine; B: British taurine; C: continental European taurine; MTN: Montana composite

Genetic structure and differentiation were observed among biological types and the Montana composite, as indicated by pairwise F_ST_ values (Fig. 9). Among biological types, the highest genetic differentiation was observed between type N and taurine types, with values ranging from 0.168 to 0.246. In relation to the Montana composite, F_ST_ values ranged from 0.022 (A – MTN) to 0.115 (N – MTN).

**Fig. 10:**
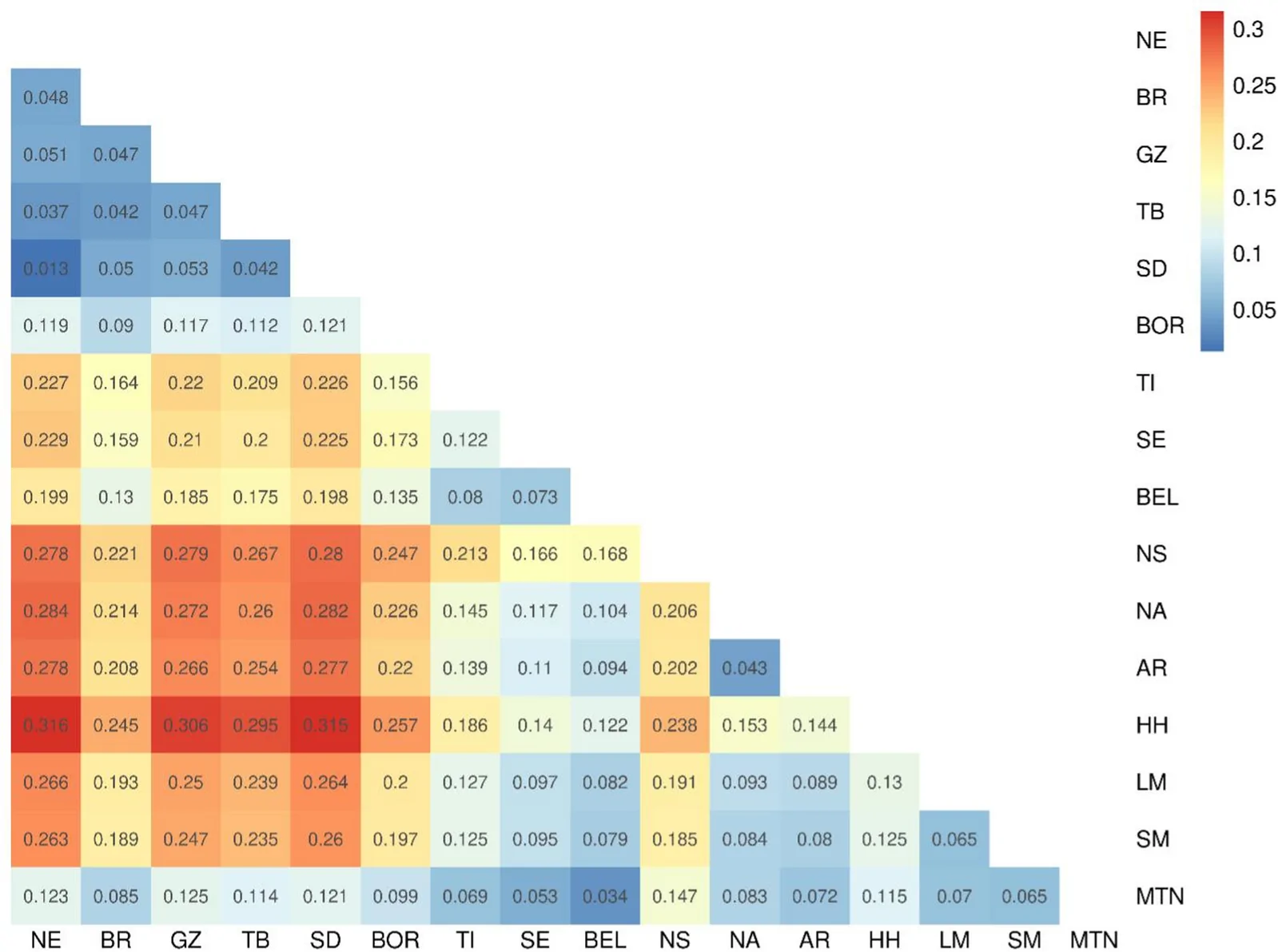
Heatmap of genetic relationships among different breeds and the Montana composite, estimated from pairwise F_ST_ values. NE: Nellore; BR: Brahman; GZ: Guzerat; TB: Tabapua; SD: Sindi; BOR: Boran; TI: Tuli; SE: Senepol; BEL: Belmont Red; NS: Bonsmara; NA: Aberdeen Angus; AR: Red Angus; HH: Hereford; LM: Limousin; SM: Simmental; MTN: Montana composite

Similarly, the analysis at the breed level (Fig. 10) showed that the lowest genetic differentiation generally occurred among breeds belonging to the same biological type, whereas the greatest genetic distances were observed between Zebu and taurine breeds. Values above 0.15 observed between Zebu and taurine breeds indicate high genetic differentiation. Within biological types, F_ST_ values ranged from 0.013 to 0.121 for group N, from 0.073 to 0.213 for group A, and from 0.043 to 0.144 for group B. The lowest genetic differentiation was observed between Sindi and Nellore (0.013), indicating high genetic proximity within the Zebu group, while the highest differentiation occurred between Nellore and Hereford (0.316), reflecting the high level of divergence between Bos indicus and Bos taurus breeds. In relation to the Montana composite, values ranged from 0.034 (Belmont Red) to 0.147 (Bonsmara).

## Discussions

### Principal component analysis

The PCA plots (Fig. 3) showed the genomic population structure of the composite population and its main founder breeds. The selection of SNPs used proved to be informative for capturing patterns of population structure and genetic differentiation, revealing a clear separation between Zebu and taurine groups along PC1, as well as partial overlap among genetically closer populations. Montana composite animals showed a wide dispersion along the two components (Figs. 3a and b), but with greater relative proximity to taurine groups along PC1, corroborating the results of Santos et al. (2025). This greater proximity of the Montana composite to taurine breeds was expected, considering the criteria defined by the International Montana Breeders Association for animal classification. According to these criteria, a maximum of 6/16 contribution from group N is allowed, whereas the maximum limits for taurine groups are 14/16 for type A and 12/16 for types B and C.

In addition, the breeds Tuli, Belmont Red, and Bonsmara, belonging to biological type A, were positioned closer to the composite. This pattern may be attributed to the genetic background of these breeds, which involves historical contributions from hybrid populations of the Sanga group, formed from crosses between African *Bos taurus* and *Bos indicus* around the 7th century AD (Hanotte et al. 2000). The Senepol breed also showed genetic proximity to the composite, which may be related to the process of selection directed toward adaptation to tropical environments. This shared adaptation may contribute to the genomic similarity observed between this breed and the composite population adapted to tropical conditions, such as Montana.

Within the breeds considered in biological type N, part of the Brahman population showed relative proximity to the composite, which may be related to its historical formation, involving selection processes and crosses among different populations, potentially resulting in reduced levels of taurine introgression (Briggs and Briggs 1980). Results based on genomic analyses have confirmed the presence of taurine allele introgression in the Brahman breed and have shown that only a small proportion of the genome exhibits complete fixation of indicine origin (Koufariotis et al. 2018). This historical introgression may also contribute to the observed proximity between Brahman and the composite.

A potential limitation of this study is the reduction in marker density to the 17,619 SNPs shared across the genotyping platforms, as well as the partial confounding between specific breeds and genotyping arrays. However, the quality control and informativeness metrics (Table S1) indicated that the retained markers provided adequate genetic variation across both *Bos taurus* and *Bos indicus* populations. Furthermore, the patterns observed in PCA and F_ST_ were consistent with the expected genetic relationships among the evaluated breeds.

### Admixture analysis

The supervised admixture analyses demonstrated that the inferred genomic composition of the Montana composite varied according to the number of ancestral populations considered. Despite differences in ancestry resolution among the evaluated models, all values of K consistently showed the predominance of biological type A and the reduced contribution of type N. These findings corroborate those of Oliveira et al. (2024), who, based on pedigree records, reported a progressive increase in the participation of type A and a reduction in the contribution of type N across birth years in the Montana composite. According to the authors, these changes were associated with selection for improved carcass composition and meeting international market demands, without compromising adaptability.

Although type N, particularly Nellore, played an important role during the initial development of the composite population, its average genomic contribution was lower, which is consistent with the limits established by the breeders’ association. In addition, successive recombination over generations promotes the fragmentation of ancestral blocks in hybrid populations, reducing the average size of these segments and redistributing ancestry along the genome (Janzen Nolte and Traulsen 2018). This process can contribute to the genomic mosaic observed in composite populations, but recombination itself does not reduce the overall proportion of ancestry inherited from a founder population. Therefore, the lower contribution of type N is more likely related to the historical changes in breed composition and subsequent selection and mating practices.

A comparison among the supervised admixture models showed that the choice of K substantially influenced the interpretation of breed composition. Although the K = 4 model reflects the traditional biological classification adopted by the Montana breeding program, it consistently assigned a greater proportion of ancestry to the type C than the models with K = 9 and 11. When founder breeds were evaluated individually, part of the ancestry previously attributed to the continental taurine component was redistributed mainly to Belmont Red, Bonsmara, Senepol, and Tuli, indicating that grouping genetically heterogeneous founder breeds into broad biological categories may mask their realized genomic contribution.

This pattern is consistent with the assumptions of the admixture model, in which individuals are represented as mixtures of K ancestral populations and ancestry proportions are estimated conditional on the predefined number of ancestral clusters (Alexander et al. 2009). Consequently, the choice of K directly determines the level of population structure that can be resolved. As discussed by Lawson, Van Dorp, and Falush (2018), different values of K should not be interpreted as alternative representations of a single biological truth, but rather as complementary descriptions of the same underlying genetic variation at different levels of resolution. Therefore, models with lower values of K tend to capture broader population structure, whereas higher values allow the identification of finer substructure among genetically related founder populations.

The redistribution of ancestry observed in the present study is consistent with the evolutionary history of the adapted taurine breeds included in the Montana composite. Breeds such as the adapted taurine group originated through complex breeding histories involving different proportions of African taurine, European taurine, and indicine cattle (Hanotte et al. 2000). Consequently, treating these genetically heterogeneous breeds as a single ancestral population may obscure important differences among founder breeds and lead topart of their genomic contribution being absorbed by broader ancestry clusters, such as the continental taurine component.

The analysis with K = 9 and 11 revealed that, although several breeds contribute to the composition of Montana, only a few show high average contribution. This suggests that, despite the founder diversity, the targeted use of sires may have increased the genetic influence of some specific lineages. This pattern is consistent with that observed in the development of crossbred cattle such as Brangus, in which genomic architecture is reshaped over generations due to breed formation, recombination, and selection, resulting in changes in the relative representation of founder breeds in the genome of the current population (Paim et al. 2020). Thus, the historical contribution of parental breeds does not necessarily remain proportional to the realized genomic contribution in subsequent generations.

The occurrence of individuals with high ancestry assignment to a single ancestral component was observed across the different supervised admixture models evaluated. In the present population, these high ancestry profiles were associated with a limited number of highly represented paternal lineages, which may be related to unequal reproductive contribution among parental lineages and selected sires and may have influenced the genomic composition of some groups. This pattern may be related to the open structure of the Montana breeding program, in which animals from different founder backgrounds can be incorporated according to selection objectives (Ferraz et al. 1999b). Consequently, the contribution of specific lineages may become amplified over generations, leading to greater representation of particular ancestral components in subsets of the population.

The genomic complexity of the Montana composite has also been demonstrated by Santos et al. (2024), who reinforce that the genomic architecture of the Montana composite cannot be fully explained by the traditional biological type classification alone. The present study complements these findings by demonstrating that the inferred breed composition is also influenced by the resolution of the ancestry model adopted. Therefore, ancestry analyses performed at different levels of resolution provide complementary information, allowing both a broad characterization of biological types and a more detailed description of founder-breed contributions. This integrated interpretation may improve the biological characterization of composite populations and support breeding strategies based on realized genomic composition.

This complexity is further reinforced by the regional variation observed in the present study. The Montana composite was developed through continuous incorporation of diverse founder backgrounds. Consequently, differences in genomic composition among regions probably reflect the cumulative effects of sire selection and breed complementarity over successive generations. Similar patterns have been reported in composite cattle populations, where selection and breeding objectives progressively modify the realized genomic contribution of founder breeds relative to their original expected proportions (Paim et al. 2022b; Hay et al. 2022).

#### Breed composition pedigree versus genomic

Crossbred animals may present breed proportions estimated from genomic data that differ from those estimated from pedigree (Wu et al. 2020; Jaafar et al. 2022). This difference may be because pedigree provides expected proportions based on theoretical Mendelian transmission (Falconer and Mackay 1996), whereas genomic estimates capture realized inheritance (Hayes et al. 2009).

In multibreed populations, genetic structure and linkage disequilibrium patterns may vary considerably among breeds, affecting the accuracy of genomic prediction (Zhou et al. 2025). In addition, directed selection processes may increase genomic similarity among certain breeds, influencing ancestry assignment and shifting estimated proportions relative to the expected theoretical values (Wu et al. 2020). This difference may become more evident in composite populations such as Montana, which involve multiple founder breeds and successive generations of recombination, whose genomic complexity has already been described in the present study and by Santos et al. (2024).

The present results indicate that the comparison between pedigree and genomic breed composition is also influenced by the resolution of the ancestry model adopted. Using higher K values allowed the differentiation of individual founder breeds and the redistribution of ancestry previously attributed to broader biological groups into more specific components. Therefore, discrepancies between pedigree expectations and genomic estimates may reflect not only realized inheritance, but also the level of resolution used to infer genomic ancestry.

Similar discrepancies between pedigree expectations and genomic ancestry have previously been reported in the Montana composite. Using local ancestry inference, Santos et al. (2025) suggested that the complex evolutionary origin of adapted breeds and the sharing of ancestral haplotypes among founder populations could limit ancestry assignment for the four biological types. The present study extends this interpretation by showing that part of the apparent excess of continental taurine ancestry observed under low-resolution models may arise from the grouping of genetically distinct founder breeds into a single biological category. In this context, increasing the number of reference populations does not necessarily imply a biologically superior model, but rather provides a more detailed resolution of the underlying genomic structure. This highlights that ancestry estimation in composite populations is inherently model-dependent and sensitive to the definition of reference populations.

The choice of genetic model, including biological type effects and non-additive effects such as heterosis and recombination calculated from pedigree, has been shown to influence estimates of variance components and genomic predictions for economically important traits in Montana composite cattle (Almeida et al. 2025; Bis et al. 2025). If differences exist between expected and realized breed composition, the specification of breed effects and the modeling of genetic structure may be affected, with potential impacts on the estimation of variance components and the accuracy of genetic evaluations in multibreed populations (Clasen et al. 2023). Basarab et al. (2018), in crossbred herds involving Aberdeen Angus, Hereford, Charolais, and Red Angus, demonstrated that genomic indicators of retained heterozygosity and realized heterosis were significantly associated with greater productive longevity and higher lifetime production. These results indicate that genomic composition has relevant biological and economic implications.

The effect of breed composition may also be modeled using approaches alternative to the inclusion of breed or biological type proportions as covariates. Genomic models that incorporate dominance effects through locus-specific weights assigned to heterozygous genotypes allow the capture of components associated with heterosis, potentially increasing prediction accuracy and reducing bias by accounting for additional sources of phenotypic variation (Xiang et al. 2018; Liu et al. 2022), which are not fully described by average breed proportions. Furthermore, methodologies based on the inference of the Breed of Origin of Alleles (BOA) allow modeling breed contributions along the genome, considering that crossbred individuals present a mosaic of segments from different ancestral origins (Zayas et al. 2024).

Thus, it becomes relevant to investigate whether the incorporation of biological type proportions, heterosis, and recombination estimated from genomic data may improve the accuracy of genetic evaluations in composite populations. Future studies should investigate whether genomic breed proportions inferred under different ancestry resolutions differentially affect estimates of genetic parameters and the accuracy of genomic prediction for economically important traits in composite populations.

### Genetic relationship and differentiation

F_ST_ values confirm the strong genetic divergence between Zebu and taurine groups, reflecting the historical evolutionary separation between *Bos indicus* and *Bos taurus*, confirming the PCA analysis and agreeing with other studies (Verdugo et al. 2019; Gebrehiwot et al. 2020; Santos et al. 2025). In general, lower F_ST_ estimates were observed among breeds belonging to the same biological type, although some breeds within the same type, particularly type A, showed relatively higher levels of differentiation. This pattern may be related to differences in the genetic background and breeding history of these breeds, which may share adaptive characteristics without necessarily having similar genomic backgrounds. Similarly, Campos et al. (2017) observed moderate differentiation among adapted taurine breeds, despite their common classification as locally adapted populations, while lower differentiation was observed among *Bos indicus* breeds, which was attributed to gene flow among these breeds and the common origin of some of the analyzed populations.

The low genetic differentiation observed between Sindi and Nellore may be related not only to the common origin of these breeds in the Indian subcontinent but also to the recent history of the Sindi breed in Brazil. Genealogical records of the Sindi breed in Brazil were interrupted in the 1970s and resumed only in the early 2000s, during which the population remained restricted to a few conservation nuclei (Fundação Joaquim Nabuco 2019). This process resulted in a strong reduction in effective population size and in the number of breed founders, as demonstrated by pedigree-based analyses (Santana Jr et al. 2016). This context may have favored the introduction of new founders or even genetic introgression from other Zebu breeds widely used in the country, such as Nellore, contributing to the low genetic differentiation observed between these populations.

The lower genetic differentiation between Belmont Red and Montana suggests greater sharing of ancestry, which may indicate a more relevant participation of this breed in the genetic base of the population, resulting in greater similarity in allele frequencies between populations. In contrast, the higher genetic differentiation observed between Bonsmara and Montana, despite the proximity evidenced in PCA analyses, may be explained by conceptual differences between the methodologies used and by unbalanced samples. While PCA reduces the dimensionality of genomic data by preserving covariance and tends to group individuals with shared ancestry (Elhaik 2022), F_ST_ directly quantifies divergence in allele frequencies between populations (Jakobsson et al. 2013). This statistic is associated with the variance of allele frequencies among populations and, inversely, with the degree of genetic similarity among individuals within each population (Holsinger and Weir 2009).

Bonsmara is a composite breed formed from crosses involving approximately 5/8 Afrikaner, a breed belonging to the Sanga group, and 3/8 Hereford and Shorthorn (Bonsma 1980). Genomic studies indicate that this breed presents moderate genetic diversity, with higher allelic counts and lower linkage disequilibrium compared to Afrikaner, a pattern attributed to its composite origin (Makina et al. 2014). In addition, population structure analyses show that, despite shared ancestry, Bonsmara forms distinct genetic clusters and may present substructure associated with specific lineages (Alberts et al. 2026). Differences in allele frequencies and possible population structure effects may contribute to higher F_ST_ values in relation to the Montana composite, even when proximity is observed in PCA.

## Conclusions

The integrated analysis showed that the Montana composite presents a complex genetic structure, with clear divergence between Zebu and taurine bases and predominance of taurine ancestry in the current genomic composition. The unequal contribution of founder breeds reflects its multibreed origin and the different genetic backgrounds represented in the population. In addition, the inferred genomic composition varied according to the ancestry model adopted, indicating that different levels of resolution provide complementary information on the genetic architecture of the composite. Differences between pedigree and genomic-based estimates indicate that realized breed composition may diverge from expected values. Overall, the results provide a detailed characterization of the genomic composition and population structure of the Montana composite.

## Supplementary Information

Below is the link to the electronic supplementary material.

Supplementary Material 1

## Data Availability

The datasets generated and/or analysed during the current study are not publicly available because they are the property of the University of São Paulo, the International Montana Breeders Association, the Animal Genetics and Breeding Program, and the Neogen Corporation, and are subject to commercial and confidentiality restrictions. For scientific research purposes, the data requests should be forwarded along with the research proposal to José Bento Sterman Ferraz (jbferraz@usp.br), Fernando Baldi (fernandobaldiuy@gmail.com), Gabriela Giacomini (gabriela@montana.org.br) and Victor Breno Pedrosa (vpedrosa@neogen.com).
