# Supplementary material for: Genomic analysis of breed composition and population structure in Montana composite cattle
Source: Mamm Genome. 2026 Sep 7;37(1):106. doi: 10.1007/s00335-026-10275-8 (PMC13550078; doi:10.1007/s00335-026-10275-8)
Supplement: Supplementary file 1 [file 335_2026_10275_MOESM1_ESM.docx]

**SUPPLEMENTARY MATERIAL**

**Table S1** Mean minor allele frequency (MAF) and expected heterozygosity for each breed included in the study

| Breed | Mean minor allele frequency | Expected heterozygosity |
| --- | --- | --- |
| Nellore | 0.2541 | 0.1629 |
| Brahman | 0.2928 | 0.1867 |
| Guzerat | 0.2450 | 0.1597 |
| Tabapua | 0.2541 | 0.1654 |
| Sindi | 0.2537 | 0.1634 |
| Boran | 0.2682 | 0.1751 |
| Tuli | 0.3018 | 0.1945 |
| Belmont Red | 0.3485 | 0.2157 |
| Bonsmara | 0.2910 | 0.1878 |
| Senepol | 0.3467 | 0.2149 |
| Aberdeen Angus | 0.3237 | 0.2016 |
| Red Angus | 0.3264 | 0.2036 |
| Hereford | 0.2906 | 0.1875 |
| Limousin | 0.3346 | 0.2082 |
| Simmental | 0.3386 | 0.2102 |

**Fig. S1** Cross-validation error of admixture analyses for K values ranging from 1 to 25

**Fig. S2** Ancestry proportions of Montana composite animals estimated by supervised admixture analysis (K = 4). Individuals are ordered according to their predominant ancestry component: (a) N: Zebu, (b) A: adapted taurine, (c) B: British taurine; (d) C:continental European taurine

**Fig. S3** Ancestry proportions of Montana composite animals estimated by supervised admixture analysis (K = 9). Individuals are ordered according to their predominant ancestry population. The population shown correspond to: (a) grouped indicine breeds (Nellore, Brahman, Guzerat, Tabapua and Sindi), (b) Boran, (c) Tuli, (d) Belmont Red, (e) Bonsmara, (f) Senepol, (g) British taurine, (h) Limousin, and (i) Simmental

**Fig. S4** Ancestry proportions of Montana composite animals estimated by supervised admixture analysis (K = 9). Individuals are ordered according to their predominant ancestry population. The population shown correspond to: (a) grouped indicine breeds (Nellore, Brahman, Guzerat, Tabapua and Sindi), (b) Boran, (c) Tuli, (d) Belmont Red, (e) Bonsmara, (f) Senepol, (g) Aberdeen Angus, (h) Red Angus, (i) Hereford, (j) Limousin, and (k) Simmental

**Table S2** Statistical comparison between pedigree-based and genomic ancestry proportions for biological types at K = 4, 9, and 11

| K | Type | W | P-value | FDR-adjusted P | Effect size | 95% CI |
| --- | --- | --- | --- | --- | --- | --- |
| 4 | N | 6669469 | <0.001 | <0.001 | 0.8364 | [0.8249 \| 0.8471] |
|  | A | 5494718 | <0.001 | <0.001 | 0.5129 | [0.4853 \| 0.5395] |
|  | B | 3690362 | 0.3893 | 0.3893 | 0.0161 | [-0.0208 \| 0.0530] |
|  | C | 1650630 | <0.001 | <0.001 | -0.5455 | [-0.5720 \| -0.5179] |
| 9 | N | 5816712 | <0.001 | <0.001 | 0.6016 | [0.5775 \| 0.6246] |
|  | A | 3138942 | <0.001 | <0.001 | -0.1357 | [-0.1716 \| -0.0995] |
|  | B | 6508556 | <0.001 | <0.001 | 0.7921 | [0.7779 \| 0.8054] |
|  | C | 2155022 | <0.001 | <0.001 | -0.4066 | [-0.4382 \| -0.3740] |
| 11 | N | 5887034 | <0.001 | <0.001 | 0.6209 | [0.5977 \| 0.6431] |
|  | A | 3317652 | <0.001 | <0.001 | -0.0865 | [-0.1228 \| -0.0500] |
|  | B | 5398864 | <0.001 | <0.001 | 0.4865 | [0.4578 \| 0.5142] |
|  | C | 3058576 | <0.001 | <0.001 | -0.1579 | [-0.1951 \| -0.1201] |

W: Wilcoxon signed-rank test statistic; FDR-adjusted P: P-value adjusted using the Benjamini–Hochberg procedure; Effect size: rank-biserial correlation (r); 95% CI: 95% confidence interval.

**Table S3** Comparison of pedigree and genomic-based estimates of breed composition in the Montana composite population across admixture analysis (K = 9 and K = 11)

| Breed / Group | Pedigree | K = 9 | K = 11 |
| --- | --- | --- | --- |
| Grouped indicine breeds | 0.2023 | 0.0708 | 0.0781 |
| Boran | 0.0002 | 0.0653 | 0.0600 |
| Tuli | 0.0170 | 0.1205 | 0.0992 |
| Belmont Red | 0.0265 | 0.1094 | 0.1722 |
| Bonsmara | 0.2189 | 0.1521 | 0.1101 |
| Senepol | 0.2030 | 0.1444 | 0.1355 |
| British taurine | 0.1634 | 0.1323 | - |
| Aberdeen Angus | 0.0163 | - | 0.0300 |
| Angus Red | 0.1314 | - | 0.1172 |
| Hereford | 0.0157 | - | 0.0199 |
| Limousin | 0.0162 | 0.1112 | 0.1078 |
| Simmental | 0.0304 | 0.0886 | 0.0699 |
